# Identification of metabolism-related genes for predicting peritoneal metastasis in patients with gastric cancer

**DOI:** 10.1186/s12863-022-01096-0

**Published:** 2022-12-12

**Authors:** Chenyu Tian, Junjie Zhao, Dan Liu, Jie Sun, Chengbo Ji, Quan Jiang, Haojie Li, Xuefei Wang, Yihong Sun

**Affiliations:** Department of General Surgery, Zhongshan Hospital, Fudan University, Shanghai, China

**Keywords:** Gastric cancer, Metabolism, Peritoneal metastasis, Gene

## Abstract

**Objective:**

The reprogramming of metabolism is an important factor in the metastatic process of cancer. In our study, we intended to investigate the predictive value of metabolism-related genes (MRGs) in recurrent gastric cancer (GC) patients with peritoneal metastasis.

**Methods:**

The sequencing data of mRNA of GC patients were obtained from Asian Cancer Research Group (ACRG) and the GEO databases (GSE53276). The differentially expressed MRGs (DE-MRGs) between a cell line without peritoneal metastasis (HSC60) and one with peritoneal metastasis (60As6) were analyzed with the Limma package. According to the LASSO regression, eight MRGs were identified as crucially related to peritoneal seeding recurrence in patients. Then, disease free survival related genes were screened using Cox regression, and a promising prognostic model was constructed based on 8 MRGs. We trained and verified it in two independent cohort.

**Results:**

We confirmed 713 DE-MRGs and the enriched pathways. Pathway analysis found that the MRG-related pathways were related to tumor metabolism development. With the help of Kaplan–Meier analysis, we found that the group with higher risk scores had worse rates of peritoneal seeding recurrence than the group with lower scores in the cohorts.

**Conclusions:**

This study developed an eight-gene signature correlated with metabolism that could predict peritoneal seeding recurrence for GC patients. This signature could be a promising prognostic model, providing better strategy in treatment.

**Supplementary Information:**

The online version contains supplementary material available at 10.1186/s12863-022-01096-0.

## Introduction

Gastric cancer (GC) is the fifth most common cancer and the third most common cause of cancer-related death globally [1]. Gastric cancer is also one of the most common cancers in China. According to 2015 statistics, in China, there were nearly 679,100 new cases of GC and 498,000 new mortalities due to GC [2]. Peritoneal metastasis (PM) is very common in gastric cancer and occurs in 53%-66% of patients with distant metastatic gastric cancer [3]. The median survival time is only 4 months once peritoneal metastasis is diagnosed, compared with 14 months in GC patients without peritoneal metastasis [4]. Serosal invasion is the strongest indicator of peritoneal metastasis in GC [5]. Most patients with GC with serosal invasion succumb to peritoneal metastasis within 2 years after surgery despite radical gastrectomy [6]. Therefore, it is quite significant to search for biomarkers that can effectively predict postoperative peritoneal metastasis and recurrence in patients with gastric cancer.

There are many pathways associated with peritoneal metastasis of a tumor. Various molecules and related signaling pathways play important roles in this process. PRL-3 downregulates PTEN, induces PTEN phosphorylation, activates the PI3K/Akt signaling pathway, and upregulates MMP-2 and MMP-9 to promote the peritoneal metastasis of gastric cancer [7]. TGF-α secreted by stromal fibroblasts in turn promotes peritoneal metastasis of ovarian cancer through epidermal growth factor receptor (EGFR) signaling [8]. Metabolic pathways also play a significant role in tumor metastasis. The adipokine apelin promotes ovarian cancer metastasis and progression through its receptor APJ, which regulates cell proliferation, energy metabolism, and angiogenesis [9]. For gastric cancer, there are few reports on the metabolic pathways involved in peritoneal metastasis of gastric cancer, and the relationship is not yet clear. However, according to current relevant studies, various pathways involved in fat metabolism have a certain correlation with peritoneal metastasis of gastric cancer. Peritoneum-derived adipocytes induce robust lipid droplet (LD) accumulation and fatty acid oxidation in GC cells through the transcriptional upregulation of DGAT2 in a C/EBPα-dependent manner and prevent anoikis during peritoneal dissemination [10]. This finding also illustrates that lipid metabolism pathways may play a role in peritoneal metastasis of gastric cancer. Therefore, we aimed to identify the metabolic indices that could predict peritoneal metastasis of gastric cancer by analyzing the metabolic pathways and important differentially expressed genes related to peritoneal metastasis of gastric cancer. The samples with strong ability of peritoneal seeding and normal samples are compared to find the metabolism related genes. Then we used these genes to make a proper model with LASSO regression to find the patients with higher risk of recurrence of peritoneal seeding.

## Materials and methods

### Data source

GSE53276, which is an 8-sample dataset, was downloaded from GEO database (https://www.ncbi.nlm.nih.gov/geo/). The GSE53276 has 2 sets of HSC60 (*n* = 2, total 4) and 2 sets of 60As6 (*n* = 2, total 4). The 60As6 which had strong ability to cause peritoneal seeding is a cell line established from HSC60.The clinical data and RNA-sequencing datasets of patients in the ACRG cohort were obtained for training. The clinical information table for this cohort is in Supplementary Table 1. The date of recurrence and the first site of recurrence were used. The RNA-sequencing datasets of GC patients in the Zhongshan cohort were obtained for validation. All patients were treated with surgery and chemotherapy. The clinical information table for this cohort is in Supplementary Table 2.

### Selection of metabolism-related genes

In our study, We downloaded MRGs from the website of GeneCards (https://www.genecards.org/) with the key words “metabolism”. Correlation scores were used to indicate the strength of the correlation between genes and metabolism. The higher score represents a stronger relationship. Follow-up studies and analyses were performed for MRGs with scores above 5.

### Differentially expressed MRGs, GO and KEGG analysis

In this study, Limma R package (FDR < 0.05, ∣logFC∣ > 1) was employed to identify differentially expressed MRGs in GC mRNA expression data. The visualization of the results (such as volcano map and heat map) could be done with the help of ggplot2 and pheatmap R packages. We analyzed and visualized functional annotations for MRGs using the GOplot R package. In addition, KEGG analysis was also performed [11–13].

### Establishment of an individual prognostic indicators according to MRG

Combined with clinical data, MRGs mRNA expression levels were analyzed. Important MRGs were selected by univariate Cox regression analysis. Genes identified as associated with peritoneal seeding recurrence time were used to develop polygenic prognostic characteristics. Multivariate MRG model was constructed by LASSO regression method using glmnet R package (11,12). In the LASSO regression, only genes with non-zero coefficients and *p* < 0.05 were selected for further calculation of risk scores (13).

According to the coefficients from LASSO regression analysis, the formula of Risk Score (RS) was built. Median RS was selected as the cut-off value, and the ACRG patients were divided into high-risk and low-risk subgroups, respectively. Kaplan Meier curve was drawn. The difference of peritoneal seeding recurrence time between high risk group and low risk group was assessed. The ROC curve was drawn by pROC package, and the clinical significance of these identified genes was evaluated.

### Statistical analysis

This study used R software (version 3.6.1) to perform statistical analysis and visualization. log-rank test and the Kaplan–Meier curve was employed to test for significant differences in Recurrence Free Survival (RFS) between the groups. Univariate and multivariate Cox proportional hazards regression analyses were also used to evaluate the relationship between the risk score and RFS. In all analyses, a *P* value < 0.05 was considered statistically significant.

## Results

### Identification of DE-MRGs

**Fig. 1:**
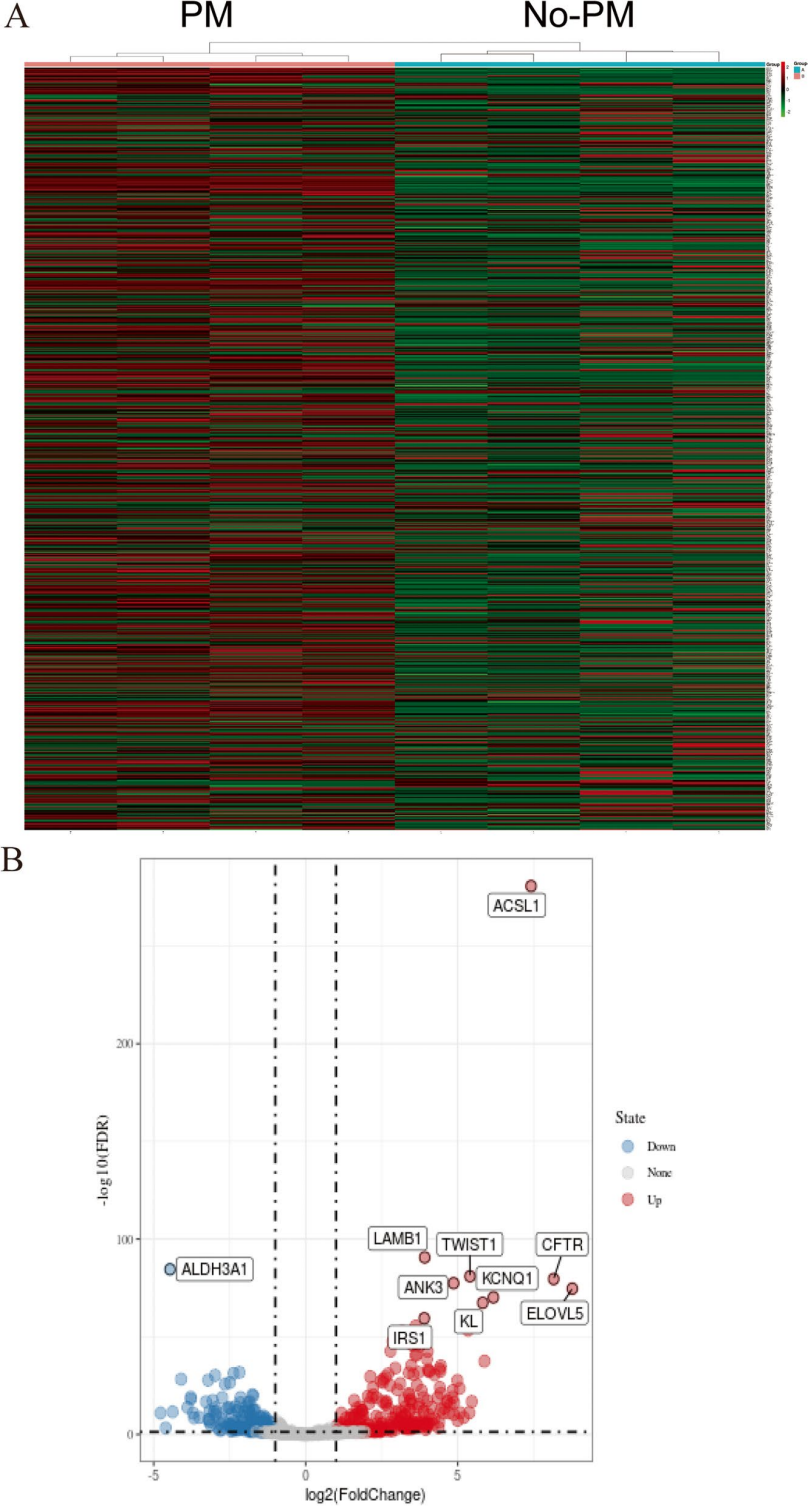
Differentially expressed MRGs between a highly peritoneal-metastatic GC cell line and a nonperitoneal-metastatic GC cell line. (A) Heatmap of the MRGs from the GSE53276 dataset; (B) volcano plot of the screened MRGs

The RNA-seq data of a pair of highly peritoneal-metastatic gastric cancer cell lines and no peritoneal-metastatic gastric cancer cell lines(GSE53276)were downloaded and analyzed. We acquired 2436 MRGs with scores > 5. The expression levels were compared of 2436 MRGs in the RNA-seq data from gastric cancer cell lines and highly peritoneal-metastatic gastric cancer cell lines. The results revealed a total of 713 significantly differentially expressed MRGs (Supplementary Table 3), including 490 significantly upregulated genes and 223 significantly downregulated genes in PMGC (Fig. 1A and 1B).

### Important pathways involved with MRGs

**Fig. 2:**
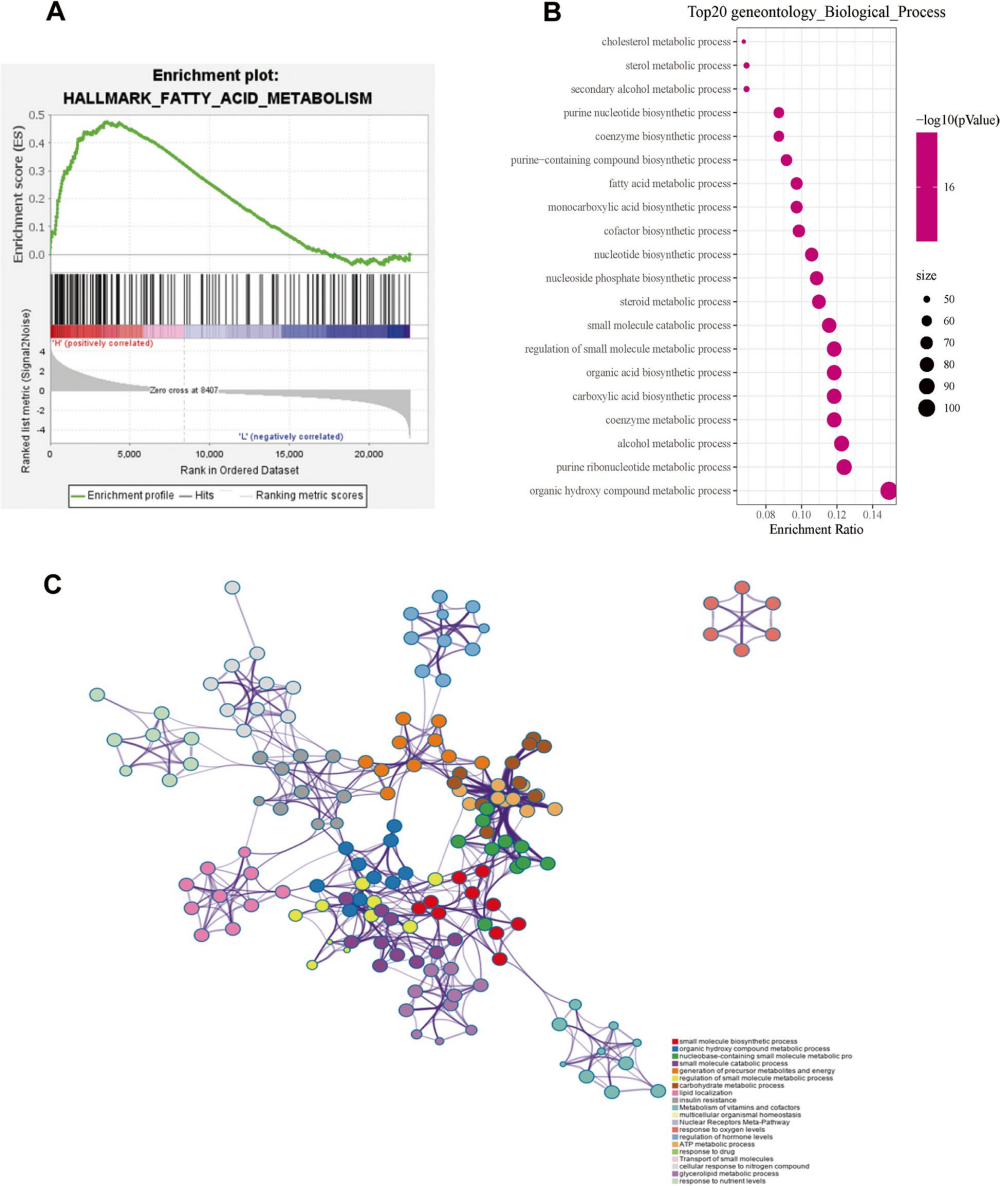
Gene set enrichment analysis and Gene Ontology analysis of differentially expressed MRGs. (A) Gene set enrichment analysis of differentially expressed MRGs. (B) Significantly enriched Gene Ontology (GO) terms of the differentially expressed MRGs according to biological processes. (C) Significantly enriched GO pathways of the DE-MRGs

**Fig. 3:**
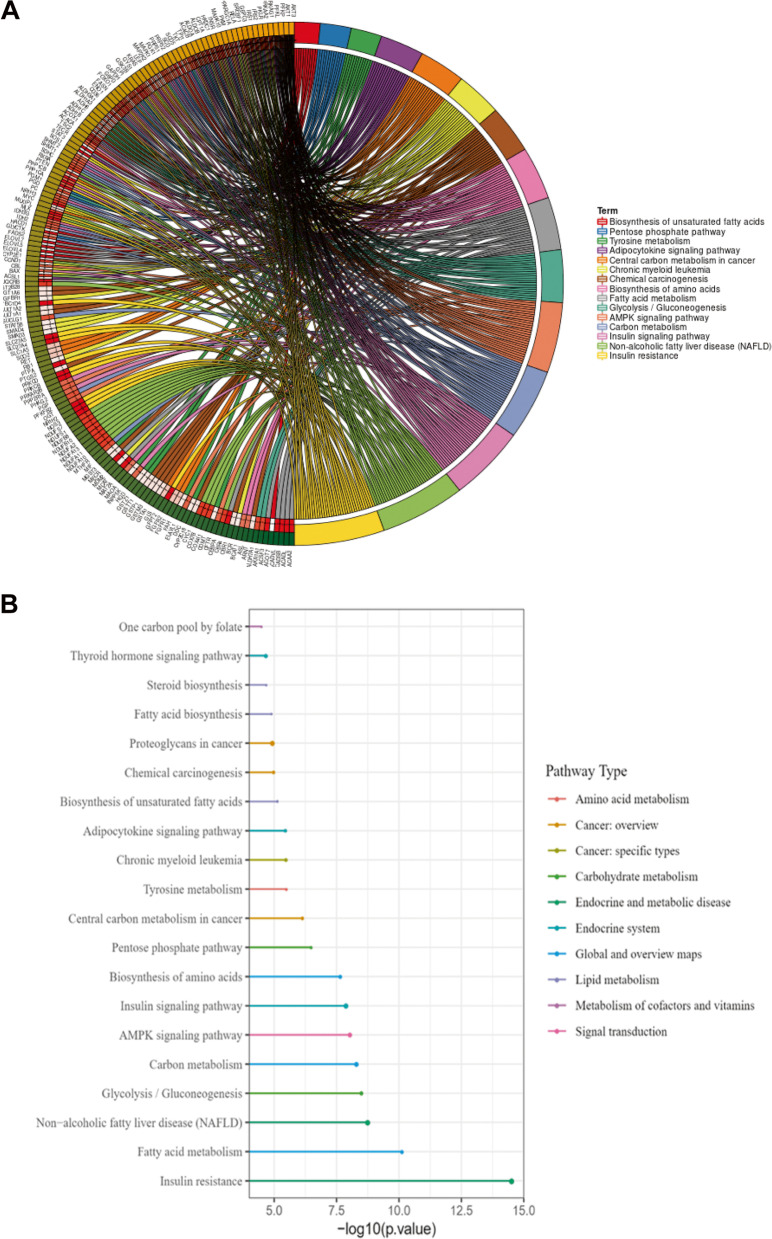
Kyoto Encyclopedia of Genes and Genomes (KEGG) analysis of differentially expressed MRGs. (A) Chord plot indicates the relationships between the genes and KEGG pathways. (B) Significantly enriched KEGG pathways of the DE-MRGs

The significant pathways of 713 differentially expressed MRGs were analyzed. GSEA found that fatty acid metabolism was one of the most important pathways (Fig. 2A). Fatty acid metabolism was also found to be an important part of the GO analysis and KEGG analysis. GO enrichment analysis showed that the differentially expressed MRGs played an important role in small molecule biosynthesis, fatty acid metabolism pathways and other metabolic pathways in GC (Fig. 2B, and 2C). KEGG analysis revealed that the DE-MRGs mainly participated in tumor metabolism related pathways, including insulin metabolism and fatty acid metabolism (Fig. 3A, and 3B).

### Construction of the MRG-based prognostic model

**Fig. 4:**
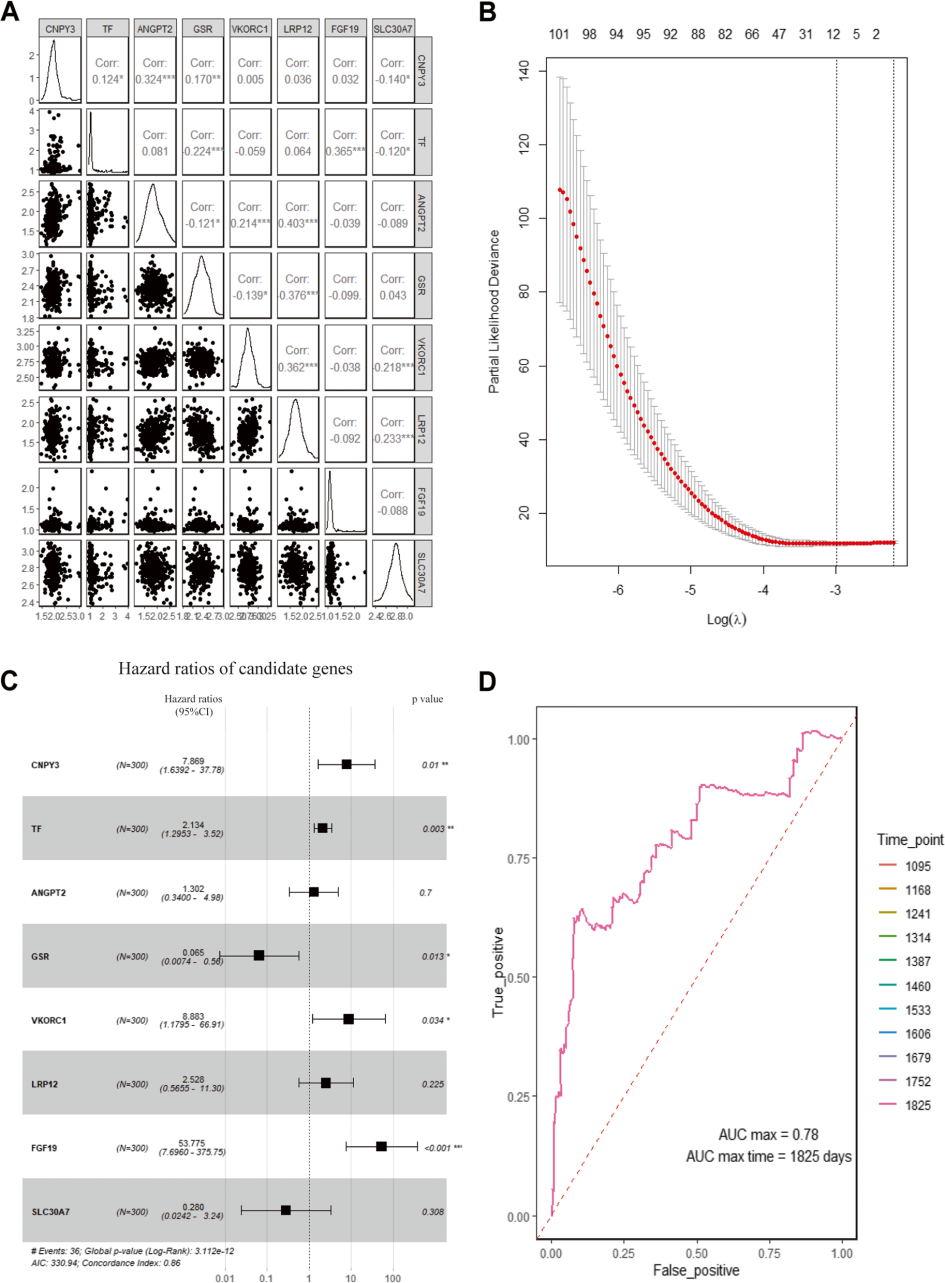
Establishment of the prognostic model for GC via LASSO regression analysis. (A) Correlation coefficients of the candidate genes. (B) A coefficient profile plot was generated against the log (lambda) sequence. (C) Univariate Cox regression analysis of the candidate genes. (D) ROC curve of the prediction model

This study used univariate Cox regression analysis to determine correlations among 713 differentially expressed MRGs and peritoneal metastasis of gastric cancer in the ACRG cohort. The results showed that 87 genes were significantly correlated with the PM related RFS of GC patients (P < 0.05). LASSO Cox regression analysis was implemented on 87 significant genes, and 8 genes (CNPY3, TF, ANGPT2, GSR, VKORC1, LRP12, FGF19 and SLC30A7) that could be independent prognostic predictors in PMGC were screened (Fig. 4A, and 4B). Based on the results of LASSO regression, the calculating formula used to get the MRG-based prognostic risk score was as follows:RS = 0.002087876 ∗ CNPY3 + 0.521922313 ∗ TF + 0.183620936 ∗ ANGPT2-0.123548398 ∗ GSR + 0.185889325 ∗ VKORC1 + 0.155022512 ∗ LRP12 + 2.808294327 ∗ FGF19 + 0.085701 ∗ SLC30A7.

In addition, multivariate Cox analysis was employed on the ACRG cohorts to make further training of the RS with GC patients (Fig. 4C, and 4D). The AUC was 0.78, which indicated that this model had an effective value for predicting the recurrence of gastric cancer patients with peritoneal metastasis.

### Training and validation of the RS model and illustration for clinical practice

**Fig. 5:**
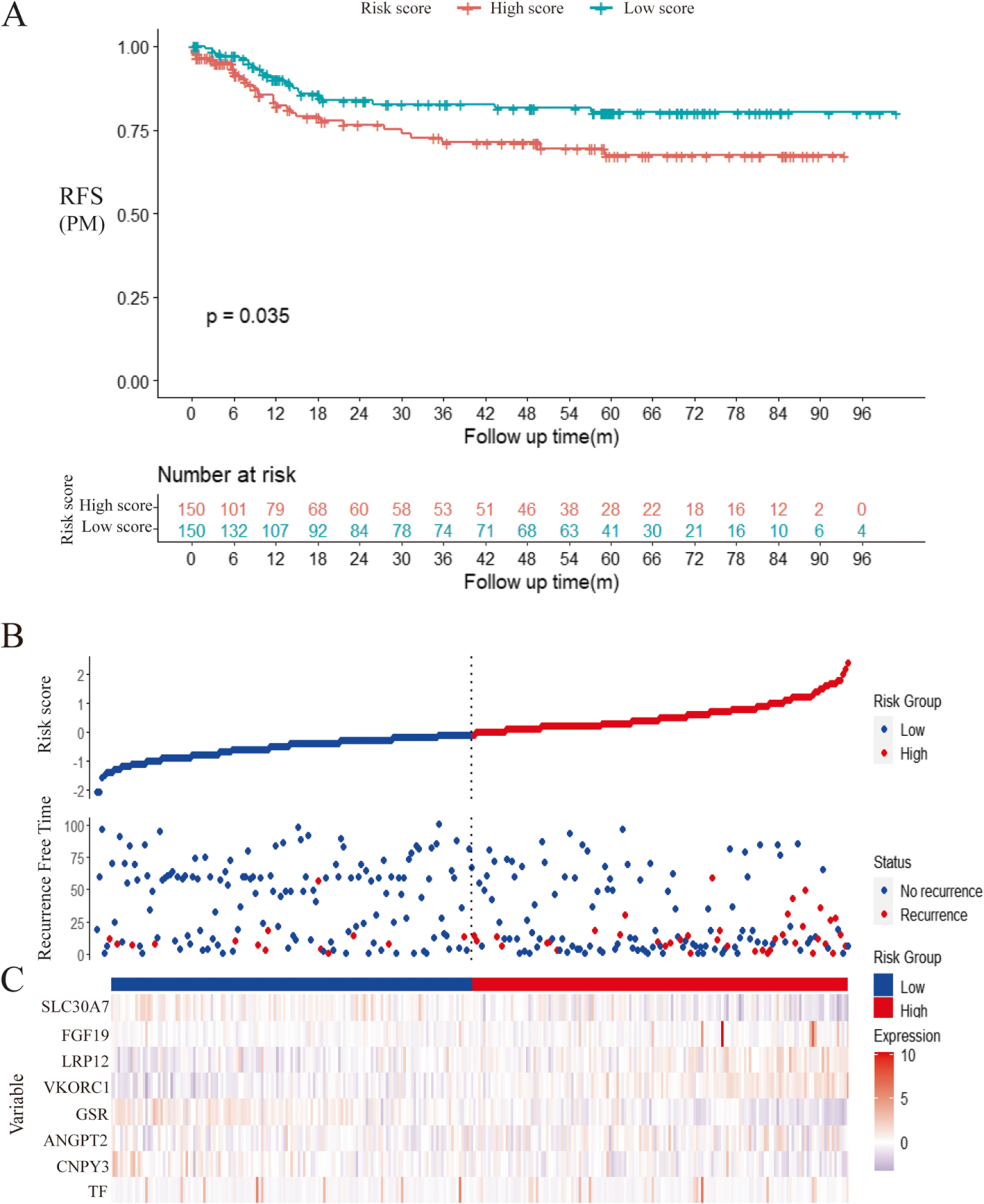
Training and clinical use of the risk score based on the 8-MRG signature in ACRG cohort. (A) Kaplan–Meier curve of the group with high risk and the group with low risk in the ACRG cohort according to RS model. (B) The RS distribution and vital status of patients. (C)The heatmap of the 8 MRG expression profiles between the high-risk group and low-risk group

**Fig. 6:**
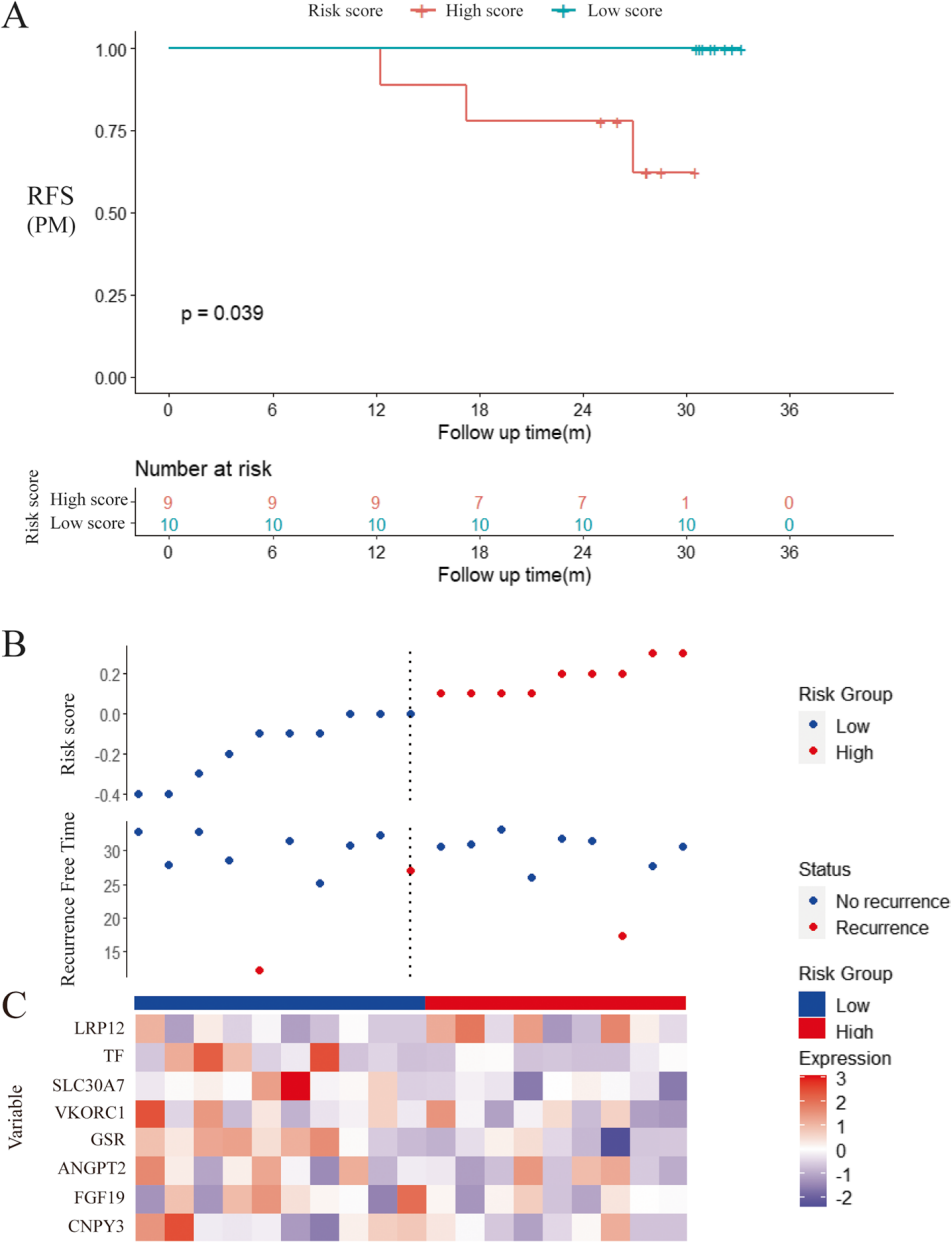
Validation and clinical use of the risk score based on the 8-MRG signature in Zhongshan GC cohort. (A) Kaplan–Meier curve of the group with high risk and the group with low risk in the Zhongshan GC cohort according to RS model. (B) The RS distribution and vital status of patients. (C)The heatmap of the 8 MRG expression profiles between the high-risk group and low-risk group

This prognostic feature based on MRGs could be employed as a predictive tool to evaluate the recurrence of peritoneal seeding pattern in patients with gastric cancer. In the ACRG cohort, each patient was assigned a risk score based on the expression levels of the 8 MRGs. The median RS of all patients was used as the critical value, and all patients were divided into the group with high risk and the group with low risk. RFS results of peritoneal seeding pattern recurrence in the low-risk group patients were significantly better than the high-risk patients (Fig. 5A). The group with high risk and the group with low risk also had differences in the RS distribution (Fig. 5B). It can be seen that patients in the high-risk group have shorter RFS and a higher recurrence rate of peritoneal seeding. It could be found that high expression of LRP12 may have a stronger correlation with recurrence of peritoneal seeding in gastric cancer patients in the heatmap of the 8 MRG expression profiles (Fig. 5C). After training through the ACRG cohort, we performed validation in a separate Zhongshan cohort. By performing a sequencing-based RS patients who underwent surgery and chemotherapy, we divided the high-risk and low-risk groups. We found that patients with recurrent peritoneal metastases in the validation cohort were in the high-risk group, while no recurrent peritoneal metastases were observed in the low-risk group, suggesting that our model can predict the risk of peritoneal metastases to some extent (Fig. 6A). The group with high risk and the group with low risk also had differences in the RS distribution and heatmap (Fig. 6B, and 6C).

## Discussion

Peritoneal metastasis of tumors is usually closely related to changes in metabolic pathways. Metabolic reprogramming is required for cancer cells to shed into the peritoneal cavity to resist anoikis. Several important genes that are closely related to metabolism, such as FGF19, LRP12, CNPY3 and TF, were investigated in our study. FGF19 induces lipolysis by activating the FGFR1c receptor present mainly in adipose tissue and other tissues except the liver. On the other hand, FGF19 induces lipogenesis through the FGFR4 receptor and inhibits the hepatic synthesis of fatty acids [14]. FGF19-FGFR4 signaling is implicated in many cellular processes, including cell proliferation, migration, metabolism and differentiation [15]. FGFR4-deleted YTN16 cells fail to form tumors and show low rates of peritoneal dissemination [16]. FGF19 was significantly associated with depth of invasion, lymph node metastasis, and TNM stage in gastric cancer [17]. LRP12 shows significant associations with the saturated fatty acid content in the gluteus medius. In light of these and other findings, the potential involvement of LRP12 in muscle lipid metabolism deserves to be further explored [18]. LRP12 may also be related to fatty acid metabolism in humans. CNPY3 is required for the membrane localization of TLR2 and Paneth cell function [19]. Currently, there are few studies on the CNPY3 gene, and further studies on its correlation with fatty acid metabolism are needed. As a chaperone of FGF19, CNPY3 may assist FGF19 in playing a specific role in the process of fatty acid metabolism. Vitamin K epoxide reductase complex subunit 1 (VKORC1) plays an important role in bone development and bone metabolism by influencing the vitamin K cycle [20]. Evidence that VKORC1 is directly involved in fatty acid metabolism is not clear. TF and anoikis have been reported to have a close relationship in relevant studies. FVIIa inhibits cell death and caspase-3 activation induced at physiologically relevant concentrations by serum deprivation and loss of adhesion in TF-overexpressing cells but not in non-TF-expressing cells [21]. This result indicates that TF overexpression can exert a certain inhibitory effect on anoikis. Although there are few reports of direct evidence of transferrin and fatty acid metabolism, the relationship between fatty acid and iron metabolism is clear. An in vitro study on endothelial cells showed that iron alone had little effect; however, after it was combined with palmitic acid, iron-mediated toxicity was markedly potentiated, as reflected in mitochondrial dysfunction, cell death, apoptosis, and DNA mutation [22]. ANGPT2 plays a crucial role not only in angiogenesis but also in the uptake and storage of fatty acids. The adipocyte-specific deletion of ANGPT2 markedly reduces fatty acid uptake and storage in subcutaneous adipose tissue. Mechanistically, ANGPT2 activates integrin α5β1 signaling in the endothelium and triggers fatty acid transport via CD36 and FATP3 into subcutaneous adipose tissue [23]. ANGPT2 has been reported to be involved in several kinds of cancer, but its role in peritoneal metastasis of gastric cancer is still not fully understood. Slc30a7 (ZnT7) is a widely expressed zinc transporter involved in the sequestration of zinc into the Golgi apparatus and vesicular compartments [24]. A reduction in Znt7 expression blunts activation of the signal transduction pathways that regulates both basal and insulin-stimulated glucose uptake in adipocytes, resulting in low glucose uptake and lipid accumulation [25]. It was reported that there was an upregulation of specific ZnT and ZIP transcripts such as Znt7 in colorectal cancer [26]. Although most of these metabolism-related genes have not been reported to be associated with peritoneal metastasis in gastric cancer, some of them were strongly linked to tumor progression. Further studies on why they affect the risk of peritoneal metastasis in gastric cancer patients were needed.

Metastasis requires cells to respond to a number of biological challenges, including escaping from the primary tumor, colonization of distant organs, and growth into tumors at these remote sites [27, 28]. Many factors contribute to the ability of cancer cells to metastasize. Metastasis also imposes different metabolic demands than those that support cell growth. In the process of peritoneal metastasis in gastric cancer cells, there are also metabolic changes. These metabolic changes may not necessarily be concentrated in the expression of a single gene, but rather manifest as an overall trend change in the metabolic network. For example, the abundance of lipids on the omentum may be associated with increased expression of genes related to lipid metabolism in cells of gastric cancer patients undergoing peritoneal metastasis. The numerous metabolism-related genes may be able to make some predictions about the risk of peritoneal metastasis in gastric cancer patients.

In our work, we downloaded the data of 300 GC patients from ACRG, and the relationship between the clinical data and the expression of MRGs was analyzed. In addition, risk scores were calculated based on MRGs as independent prognostic indicators of recurrence. These results revealed some crucial MRGs in GC, which also might make a contribution for follow-up studies on tumor metabolism. Our findings could provide a useful risk scoring formula for predicting recurrence and guiding treatment for GC patient. A well-designed prediction model could facilitate communication between physicians and patients and identify patients who are genuinely at high risk. The recurrence risk score, an effective prognostic tool designed to improve the prediction of gastric cancer after diagnosis, was validated in this study. In this study, the recurrence risk score was based on the fractions of 8 important metabolism-related genes. The results showed a clear separation of overall survival curves between patients with high and low recurrence risk scores. Although some of the eight genes do not have a strong direct link to the peritoneal metastasis of cancer, the models based on these genes suggest that they might take part in tumor development and metastasis. The specific mechanism needs to be further explored in the future.

In summary, this study identified the DE-MRGs and pathways associated with the metabolic process in GC. These genes might serve as potential biomarkers to make the prediction of prognosis and diagnosis of the peritoneal metastasis of GC patients and provide new perspectives for therapy.

## Supplementary Information

**Additional file 1:** **Supplementary Table 1.** Baselinedata of 300 patients with gastric cancer in ACRG cohort. **Supplementary Table 2.** Baselinedata of 19 patients with gastric cancer in Zhongshan cohort. **Supplementary Table 3.** The list of 713 DE-MRGs. **Supplementary Table 4.** Detailedinformation on the 8 key Metabolism-Related Genes.

## Data Availability

The datasets used or analyzed during the current study are available from GEO repository: https://www.ncbi.nlm.nih.gov/geo/.
