# Supplementary material for: Identification of metabolism-related genes for predicting peritoneal metastasis in patients with gastric cancer
Source: BMC Genom Data. 2022 Dec 12;23:84. doi: 10.1186/s12863-022-01096-0 (PMC9743729; doi:10.1186/s12863-022-01096-0)
Supplement: Supplementary file 1 — Additional file 1: Supplementary Table 1. Baselinedata of 300 patients with gastric cancer in ACRG cohort. Supplementary Table 2. Baselinedata of 19 patients with gastric cancer in Zhongshan cohort. Supplementary Table 3. The list of 713 DE-MRGs. Supplementary Table 4. Detailedinformation on the 8 key Metabolism-Related Genes. [file 12863_2022_1096_MOESM1_ESM.docx]

**Supplementary Table 1** **Baseline data of 300 patients with gastric cancer in ACRG cohort**

| **Clinicopathological characteristics** | Number | Percentage (%) |
| --- | --- | --- |
| **Gender** |  |  |
| Male | 199 | 66.33 |
| Female | 101 | 33.67 |
| **Age** |  |  |
| <=65 | 172 | 57.33 |
| >65 | 128 | 42.67 |
| **T** |  |  |
| T1 | 0 | 0 |
| T2 | 188 | 62.67 |
| T3 | 91 | 30.33 |
| T4 | 21 | 7.00 |
| **N** |  |  |
| N0 | 38 | 12.67 |
| N1-3 | 262 | 87.33 |
| **M** |  |  |
| M0 | 273 | 91.00 |
| M1 | 27 | 9.00 |
| **TNM stage** |  |  |
| I | 30 | 10.00 |
| II | 97 | 32.33 |
| III | 96 | 32.00 |
| IV | 77 | 25.67 |
| **Location** |  |  |
| Antrum | 155 | 51.66 |
| Body | 107 | 35.67 |
| Cardia | 32 | 10.67 |
| Whole | 6 | 2.99 |
| **Lauren classification** |  |  |
| Intestinal | 146 | 48.67 |
| Diffuse | 134 | 44.67 |
| Mixed | 20 | 6.66 |
| **First site of recurrence** |  |  |
| Peritoneal seeding | 54 | 18.00 |
| liver | 36 | 12.00 |
| Bones | 7 | 2.33 |
| Others | 28 | 9.33 |
| No recurrence | 157 | 52.33 |
| Unknown | 18 | 6.00 |

T, Primary tumor; N, Regional lymph nodes; M, Distant metastasis; TNM, Tumor Node Metastasis.

**Supplementary Table 2** **Baseline data of 19 patients with gastric cancer in Zhongshan cohort**

| **Clinicopathological characteristics** | Number | Percentage (%) |
| --- | --- | --- |
| **Gender** |  |  |
| Male | 15 | 78.95 |
| Female | 4 | 21.05 |
| **Age** |  |  |
| <=65 | 11 | 57.89 |
| >65 | 8 | 42.11 |
| **T** |  |  |
| T1 | 0 | 0 |
| T2 | 0 | 0 |
| T3 | 3 | 15.79 |
| T4 | 16 | 84.21 |
| **N** |  |  |
| N0 | 0 | 0 |
| N1-3 | 19 | 100.00 |
| **M** |  |  |
| M0 | 19 | 100.00 |
| M1 | 0 | 0 |
| **TNM stage** |  |  |
| I | 0 | 0 |
| II | 3 | 15.79 |
| III | 16 | 84.21 |
| IV | 0 | 0 |
| **Lauren classification** |  |  |
| Intestinal | 10 | 52.63 |
| Diffuse | 4 | 21.05 |
| Mixed | 5 | 26.32 |
| **First site of recurrence** |  |  |
| Peritoneal seeding | 3 | 15.79 |
| No Recurrence | 16 | 84.21 |

T, Primary tumor; N, Regional lymph nodes; M, Distant metastasis; TNM, Tumor Node Metastasis.

**Supplementary Table 3 The list of 713 DE-MRGs**

| Genes |
| --- |
| CYP2E1 |
| SCARB1 |
| MAPK1 |
| GAMT |
| ALMS1 |
| ACACB |
| TNFRSF10A |
| FLCN |
| CNPY3 |
| MOGAT1 |
| C9orf72 |
| KLF11 |
| LACC1 |
| TFAP2B |
| HFE |
| ACSF3 |
| ITGB1 |
| SYCP3 |
| CBR4 |
| CHRM3 |
| NT5E |
| CYP19A1 |
| DYNC2LI1 |
| HACD1 |
| TK1 |
| PPA2 |
| LIG3 |
| LMNA |
| RBM8A |
| GGA1 |
| PDE4D |
| NDUFA10 |
| SLC9A1 |
| STEAP3 |
| MTHFD2L |
| CYP2U1 |
| TIA1 |
| ABCC4 |
| ACOT11 |
| FGFR4 |
| PTGS2 |
| SYNJ2 |
| STAT5B |
| ATP6V0A2 |
| NAMPT |
| C1S |
| WNK3 |
| ASXL2 |
| LRP1 |
| IFIH1 |
| PTCH1 |
| CFI |
| CFL1 |
| RHOA |
| AKR1C1 |
| DNM2 |
| CBX3 |
| SLC19A1 |
| CACNA1D |
| IGFBP5 |
| PEX13 |
| PHKG2 |
| YWHAZ |
| MGAT1 |
| DYNC1H1 |
| LRPPRC |
| GATM |
| CD44 |
| TTN |
| FN1 |
| SRC |
| KDSR |
| PGK1 |
| PECAM1 |
| TPM1 |
| MALAT1 |
| SREBF1 |
| KRAS |
| CLU |
| MCCC2 |
| PRDX5 |
| TMEM165 |
| SLC17A1 |
| PGD |
| SCN8A |
| PIEZO2 |
| INF2 |
| GPD1 |
| AMN |
| WDR72 |
| POLR3A |
| P4HB |
| ALB |
| FUS |
| NADSYN1 |
| RETSAT |
| PDXK |
| CLN6 |
| CBL |
| HNRNPC |
| BMP1 |
| LMF1 |
| SMARCA4 |
| PPARGC1A |
| PIK3C3 |
| GRIA3 |
| TECR |
| POU2AF1 |
| FMO5 |
| SERAC1 |
| MLX |
| STXBP2 |
| RPL11 |
| RPS27A |
| RPL18 |
| SLC25A3 |
| GUK1 |
| COX4I1 |
| AP2M1 |
| PFN1 |
| SOD1 |
| HDLBP |
| PHB |
| SPTBN1 |
| CD81 |
| UBE2L3 |
| GRN |
| PPP2R1A |
| PRKCSH |
| ACADVL |
| FTH1 |
| NFE2L1 |
| MAT2A |
| ACTB |
| LAMP2 |
| TPI1 |
| GSTP1 |
| PLOD1 |
| SCD |
| BCAP31 |
| PPP1CA |
| DHCR24 |
| PSMD4 |
| AHCY |
| UBA1 |
| ALDOA |
| CD59 |
| TRIM28 |
| HNRNPH1 |
| PFKP |
| CYC1 |
| DCTN1 |
| PFKL |
| TUFM |
| PGRMC1 |
| ACLY |
| IGFBP7 |
| ITPR3 |
| NDUFB8 |
| ENO1 |
| PKM |
| TOP2A |
| YME1L1 |
| CST3 |
| CSNK2B |
| MGST3 |
| PPP1CB |
| SQSTM1 |
| CTSC |
| LAMB1 |
| TGFBI |
| SRM |
| APOD |
| FHL1 |
| LAMP1 |
| GYG1 |
| KRT18 |
| MBTPS1 |
| GJA1 |
| GYS1 |
| ELAVL1 |
| LUM |
| AIP |
| RELA |
| DHCR7 |
| PRKAG1 |
| HSPB1 |
| BNIP3 |
| UBXN1 |
| IMPDH2 |
| AKR1A1 |
| COASY |
| SLC20A1 |
| ACSL1 |
| PGM1 |
| ACAA2 |
| RPL38 |
| LDLR |
| TSPO |
| COX7B |
| AP2S1 |
| AKR7A2 |
| NUP62 |
| NUP93 |
| BLVRB |
| FADS2 |
| SLC6A8 |
| STXBP1 |
| DDAH2 |
| TOMM40 |
| G6PD |
| MTHFD1 |
| BCR |
| EHMT2 |
| CFB |
| SEC24C |
| KEAP1 |
| MAP2K2 |
| RXRA |
| MYC |
| IDH3G |
| MPI |
| SMAD4 |
| DHFR |
| PRKCD |
| GSTM3 |
| FOXM1 |
| PSMB10 |
| CAD |
| IGFBP2 |
| FOXO1 |
| EBP |
| SLC7A8 |
| FBN1 |
| PIEZO1 |
| INPP5K |
| LPCAT3 |
| CLPP |
| DHPS |
| GPX2 |
| U2AF1 |
| FAH |
| ADM |
| ETFB |
| IL1R1 |
| ARNT2 |
| RNASEH2A |
| MVD |
| CYBA |
| NDUFS1 |
| HTRA2 |
| SLC11A2 |
| IMPA2 |
| SPTLC2 |
| RB1 |
| GLS |
| ALDH1A3 |
| ABCB6 |
| MTRR |
| MTMR2 |
| APRT |
| IL4R |
| SLC25A12 |
| TBC1D4 |
| TF |
| PRPS2 |
| MMD |
| POLD1 |
| MME |
| B3GAT3 |
| PSEN1 |
| GCDH |
| MPST |
| PCBD1 |
| OFD1 |
| SULT1A1 |
| CPT1A |
| FGFR2 |
| DGAT1 |
| PRKAR2B |
| DPP4 |
| AQP3 |
| TCF4 |
| ST3GAL4 |
| POLRMT |
| DMD |
| NR1H3 |
| GSTA1 |
| TAZ |
| CYP27A1 |
| CEBPA |
| PTEN |
| TPM2 |
| IL2RG |
| COL7A1 |
| PCYT1A |
| ACOT8 |
| FAAH |
| GNA11 |
| CDK2 |
| CYP11A1 |
| NAGLU |
| MAOA |
| APOC1 |
| LCAT |
| ALOX5 |
| SNCA |
| PC |
| KCNQ1 |
| UGT1A6 |
| PEX6 |
| ABCG1 |
| SLC7A7 |
| ASL |
| VCAN |
| IRS1 |
| ALDOB |
| GNAO1 |
| PEX1 |
| GMDS |
| TLR2 |
| INHBA |
| TNFRSF11B |
| TOP3A |
| MTAP |
| NF2 |
| HAGH |
| CFTR |
| XRCC4 |
| RARB |
| GFPT2 |
| CIITA |
| PLCE1 |
| ANG |
| TIMM8A |
| GALK2 |
| HGD |
| ESR1 |
| SCO2 |
| CEP290 |
| PER2 |
| KCNH2 |
| BCL10 |
| GAD1 |
| BMP2 |
| DDC |
| ACADSB |
| CFD |
| MDM2 |
| SMAD3 |
| NPAS2 |
| RYR1 |
| ANGPT2 |
| NOS3 |
| PDE9A |
| SMPD2 |
| ALDH3A1 |
| PAX6 |
| FLAD1 |
| GAL3ST1 |
| CD86 |
| CRP |
| GSR |
| SLC3A1 |
| CCK |
| UQCRB |
| RET |
| ACSM3 |
| KL |
| AK2 |
| ASPA |
| PPIL2 |
| ACADL |
| HMGA1 |
| PI4KB |
| ABCC2 |
| CETP |
| B4GALT6 |
| SPINK1 |
| ADH1C |
| NPHP1 |
| GUCY2C |
| PDK3 |
| OPA3 |
| ANK3 |
| SLC1A1 |
| PTPA |
| CD36 |
| PML |
| MGAM |
| ABAT |
| SMARCA2 |
| AKR1B10 |
| HP |
| GAD2 |
| MTHFR |
| NGF |
| CH25H |
| TGFBR1 |
| ATP2B3 |
| TNFRSF11A |
| IAPP |
| LEP |
| SULT1A2 |
| NTRK2 |
| AKT1 |
| NR1I2 |
| SLCO1A2 |
| ALG3 |
| CA5A |
| DEGS1 |
| PRSS3 |
| SLC7A11 |
| NFKB2 |
| IL4 |
| ADH6 |
| SLC28A1 |
| OGT |
| CASK |
| PEMT |
| ENTPD1 |
| NCOA3 |
| PRKAA2 |
| PTK2 |
| FGFR1 |
| MUC1 |
| INSR |
| PKLR |
| CACNA1G |
| TFR2 |
| PFKFB2 |
| PRKCB |
| GNRH1 |
| OPRM1 |
| AMPD3 |
| ACOT7 |
| CYP2C18 |
| ABCC3 |
| IFNB1 |
| GPI |
| OCRL |
| GLP1R |
| KCNJ5 |
| PRPS1 |
| RRAS2 |
| BAX |
| TUBB1 |
| SPTAN1 |
| FDFT1 |
| VCP |
| DDOST |
| BSG |
| TKT |
| CCND1 |
| OXA1L |
| ELOVL5 |
| HSPA4 |
| COMT |
| C1QBP |
| SLC1A5 |
| POR |
| PRCC |
| ALDH7A1 |
| STAT3 |
| KRT8 |
| TUBB |
| PSMB8 |
| TXN2 |
| AKR1C3 |
| MAN2B1 |
| IRS2 |
| CXCR4 |
| MPDU1 |
| CBR1 |
| SQLE |
| NDUFA2 |
| KIF1C |
| CLN3 |
| NSDHL |
| PRKD2 |
| PLPP3 |
| EPHX2 |
| DPM2 |
| SMPD1 |
| EIF2B4 |
| SYNE1 |
| RAB27A |
| GSTZ1 |
| APOL1 |
| ACOX1 |
| TST |
| ADH1B |
| RIN2 |
| CBX5 |
| NR1D2 |
| KHDRBS3 |
| SPHK2 |
| ADRA2A |
| GSK3B |
| SHMT1 |
| SLC25A1 |
| APEX1 |
| IDH2 |
| AQP4 |
| BANF1 |
| TM7SF2 |
| ITPK1 |
| GPR35 |
| GRIN2B |
| RORA |
| HYAL1 |
| AGPAT2 |
| SPINT2 |
| TXNRD2 |
| ARNT |
| AASS |
| ST3GAL6 |
| GYG2 |
| MGLL |
| CLIP2 |
| NEK1 |
| AR |
| ICOSLG |
| FSHR |
| UGT2B28 |
| NDUFS7 |
| MYH9 |
| GANAB |
| COL6A1 |
| ACACA |
| COG4 |
| OPA1 |
| FASN |
| ALDH1A1 |
| AGRN |
| NUP210 |
| NEDD4L |
| SPTSSA |
| GAPDH |
| AKT3 |
| LRBA |
| PNPLA2 |
| NME4 |
| SOS1 |
| UTRN |
| MCAT |
| CDKN1C |
| BBIP1 |
| LDB3 |
| ERCC2 |
| PHACTR1 |
| SOX2 |
| CFH |
| HLA-A |
| ZNF23 |
| TWIST1 |
| SHMT2 |
| PRDM10 |
| GAL |
| MUC5AC |
| MAPK10 |
| BCAT1 |
| SELENBP1 |
| TPH1 |
| TMPRSS6 |
| CNTNAP2 |
| GK2 |
| AGPAT1 |
| TSC2 |
| SUCLG1 |
| TIMM50 |
| SEC61A1 |
| ITM2B |
| C3 |
| TRMT112 |
| GALNT2 |
| TMEM43 |
| MIF |
| CYBRD1 |
| STUB1 |
| VKORC1 |
| DCXR |
| PTGES2 |
| MRPS34 |
| HMOX2 |
| SLC25A37 |
| SLC52A2 |
| VAC14 |
| CLDN1 |
| IP6K2 |
| OSTM1 |
| NR1H2 |
| SLC25A10 |
| MSTO1 |
| RHOT1 |
| SFXN1 |
| SNX10 |
| ALG12 |
| SLC35C1 |
| CD320 |
| DHDDS |
| ATP13A2 |
| SUV39H1 |
| MAN1B1 |
| MECR |
| PUS1 |
| ATG7 |
| SIRT7 |
| SRD5A3 |
| PANK2 |
| PLEKHA1 |
| NPR3 |
| HSD17B14 |
| UBIAD1 |
| RBP4 |
| SLC39A4 |
| CLN8 |
| DPM3 |
| PRMT7 |
| ELOVL4 |
| LRP12 |
| DCLRE1C |
| SLC27A5 |
| SLC6A14 |
| FKRP |
| ACE2 |
| SIRT4 |
| TINF2 |
| ANKH |
| L2HGDH |
| ANTXR1 |
| NPC1L1 |
| TLR7 |
| SCD5 |
| WRAP53 |
| KLF4 |
| N6AMT1 |
| PDE7B |
| HKDC1 |
| FTCD |
| CHIA |
| SLC52A1 |
| AHI1 |
| NDUFA13 |
| GFM1 |
| PDE11A |
| MLXIPL |
| NPL |
| TEX11 |
| GDF9 |
| SLC25A28 |
| HYI |
| SOAT1 |
| GDF15 |
| PEX16 |
| CHPT1 |
| NPM1 |
| G6PC3 |
| JMJD1C |
| LDLRAP1 |
| CLPB |
| CERS2 |
| PGP |
| HSD3B7 |
| LPCAT2 |
| NDUFB10 |
| FOXRED1 |
| CYP2S1 |
| TNFSF13B |
| AS3MT |
| FGF19 |
| SRA1 |
| DGAT2 |
| DNAJC5 |
| ABHD12 |
| CERS5 |
| SNX14 |
| SLC39A10 |
| MYO5B |
| NDUFA11 |
| MFSD2A |
| MTHFD1L |
| POLR3H |
| COG6 |
| SLC27A4 |
| IAH1 |
| SWI5 |
| FNDC5 |
| SLC30A7 |
| CISD2 |
| WDR81 |
| PPM1K |
| NADK2 |
| ELOVL7 |
| ALDH16A1 |
| EPG5 |
| PCSK9 |
| NMNAT3 |
| PTGR1 |
| ANO5 |
| GLIS3 |
| SGO2 |
| TRMT10A |
| ACMSD |
| SLC6A19 |
| NPHP3 |
| DEGS2 |
| SDR16C5 |
| CLYBL |
| GLYCTK |
| ARX |
| MYOT |
| RDH12 |

**Supplementary Table 4** **Detailed information on the 8 key Metabolism-Related Genes.**

| Gene | Location | Pathways involved |
| --- | --- | --- |
| FGF19 | Chromosome 11, NC_000011.10 | Signaling by Receptor Tyrosine Kinases |
|  |  | /Signaling by FGFR/etc |
| LRP12 | Chromosome 8, NC_000008.11 | Enables low-density lipoprotein particle receptor activity |
|  |  | /Enables protein binding/etc |
| CNPY3 | Chromosome 6, NC_000006.12 | Enables protein binding |
|  |  | /Enables signaling receptor binding/etc |
| ANGPT2 | Chromosome 8, NC_000008.11 | Enables metal ion binding |
|  |  | /Enables protein binding/etc |
| TF | Chromosome 3, NC_000003.12 | Enables ferric iron binding |
|  |  | /Enables ferrous iron binding/etc |
| GSR | Chromosome 8, NC_000008.11 | Enables NADP binding |
|  |  | /Enables electron transfer activity/etc |
| SLC30A7 | Chromosome 1, NC_000001.11 | Enables metal ion transmembrane transporter activity |
|  |  | /Enables zinc ion transmembrane transporter activity/etc |
| VKORC1 | Chromosome 16, NC_000016.10 | Enables protein binding |
|  |  | /Enables quinone binding/etc |

*The gene information were provided by GOA.
